# Post-Transcriptional Regulation of the Sef1 Transcription Factor Controls the Virulence of *Candida albicans* in Its Mammalian Host

**DOI:** 10.1371/journal.ppat.1002956

**Published:** 2012-11-01

**Authors:** Changbin Chen, Suzanne M. Noble

**Affiliations:** 1 Department of Microbiology and Immunology, University of California at San Francisco, San Francisco, California, United States of America; 2 Department of Medicine, Division of Infectious Diseases, University of California at San Francisco, San Francisco, California, United States of America; Carnegie Mellon University, United States of America

## Abstract

The yeast *Candida albicans* transitions between distinct lifestyles as a normal component of the human gastrointestinal microbiome and the most common agent of disseminated fungal disease. We previously identified Sef1 as a novel Cys_6_Zn_2_ DNA binding protein that plays an essential role in *C. albicans* virulence by activating the transcription of iron uptake genes in iron-poor environments such as the host bloodstream and internal organs. Conversely, in the iron-replete gastrointestinal tract, persistence as a commensal requires the transcriptional repressor Sfu1, which represses *SEF1* and genes for iron uptake. Here, we describe an unexpected, transcription-independent role for Sfu1 in the direct inhibition of Sef1 function through protein complex formation and localization in the cytoplasm, where Sef1 is destabilized. Under iron-limiting conditions, Sef1 forms an alternative complex with the putative kinase, Ssn3, resulting in its phosphorylation, nuclear localization, and transcriptional activity. Analysis of *sfu1* and *ssn3* mutants in a mammalian model of disseminated candidiasis indicates that these post-transcriptional regulatory mechanisms serve as a means for precise titration of *C. albicans* virulence.

## Author Summary

*Candida albicans* is a fungus that resides on the skin and in the gastrointestinal tract of humans and other mammals. However, this commensal organism is also capable of proliferating and causing disease in people who have received antibiotics, who are immunocompromised, or who have suffered injury to epithelial layers. We previously identified a novel transcription factor called Sef1 that promotes *C. albicans* virulence by activating the expression of iron uptake genes in iron-poor environments, such as the host bloodstream. However, in iron-replete environments such as the gastrointestinal niche, the *SEF1* gene is repressed by a second transcription factor called Sfu1. Here, we report our discovery of a series of post-transcriptional regulatory events that determine the intracellular localization, stability, and activity of Sef1 protein. Mutants that disrupt these post-transcriptional events alter *C. albicans* virulence in a mammalian model of disseminated infection. The existence of multiple levels of regulation speaks to the importance of Sef1 in *C. albicans* virulence and suggests that close titration of Sef1 activity is important for adaptation to distinct microenvironments within the mammalian host.

## Introduction

*Candida albicans* is a ubiquitous component of the mammalian microbiome [1] as well as the most common fungal pathogen of humans [2], [3], [4], [5]. As this organism transits between its commensal niches (mammalian skin and gastrointestinal tract) and those of virulence (bloodstream and internal organs), it experiences profound shifts in the levels of nutrients, the physical environment, and immune surveillance. We previously demonstrated that a novel *C. albicans* transcriptional regulatory circuit is required for survival in at least two distinct habitats, the host bloodstream and gastrointestinal tract [6], where levels of bioavailable iron differ by more than 20 orders of magnitude [7], [8]. In the bloodstream, where iron is tightly sequestered by host transferrin [7], *C. albicans* defends against iron deficiency through expression of Sef1, a Cys_6_Zn_2_ transcriptional activator of iron uptake genes and an indirect suppressor of the gene for Sfu1 [6]. In the gastrointestinal tract, where iron is abundant thanks to diet and sloughed cells [8], [9], *C. albicans* defends against iron toxicity through the expression of Sfu1 [6], a GATA family transcriptional repressor that inhibits both *SEF1* and genes for iron uptake [6], [10]. Remarkably, the opposing roles of Sef1 and Sfu1 in iron homeostasis extend to differing relationships with the host, with Sef1 promoting virulence and Sfu1 promoting commensalism in animal models [6]. However, the details of how these transcriptional regulators are themselves regulated by iron remain to be elucidated.

Sfu1 is broadly conserved among ascomycetes, and orthologs from multiple species have been shown to play a negative role in iron homeostasis through repression of iron uptake genes [10], [11], [12], [13], [14], [15]. The best-characterized ortholog is Fep1 from *Schizosaccharomyces pombe* that, like Sfu1, is subject to repression at the transcriptional level when environmental iron is limiting [16], [17]. In this species, protein activity is also regulated by iron, since only iron-bound Fep1 can associate with DNA [18]. By contrast, orthologs of Sef1 have not been extensively characterized, in part because the genomes of only a handful of species in the *Saccharomyces* and *Candida* lineages encode this protein [6]. Moreover, *C. albicans* Sef1 appears to function differently from its *S. cerevisiae* ortholog, since iron homeostasis in the latter species is controlled by Aft family proteins [19], [20], [21] and is not dependent on Sef1 [6].

Here we describe studies that reveal an unexpected, transcription-independent role of *C. albicans* Sfu1 in inhibiting Sef1 function, as well as a role for a predicted protein kinase, Ssn3, in Sef1 activation. Specifically, we find that, under iron-replete conditions, Sfu1 physically associates with Sef1 and sequesters it in the cytoplasm, where it is destabilized. In contrast, under iron-depleted conditions, Sef1 forms an alternative complex with Ssn3, resulting in Sef1 phosphorylation, nuclear localization, and the transcriptional activation of iron uptake genes. These post-transcriptional regulatory events are of direct consequence to *C. albicans* virulence, since either overexpression of *SFU1* or deletion of *SSN3* results in attenuated virulence in a mammalian model. We hypothesize that these multiple, opposing mechanisms for Sef1 regulation, including a surprising protein-protein interaction with its own transcriptional inhibitor, enable this obligate mammalian parasite to fine-tune its interactions with the host on a spectrum from commensalism to virulence.

## Results

### Iron-dependent transcriptional repression by Sfu1 accounts for the observed regulation of *SEF1*

**Figure 1:**
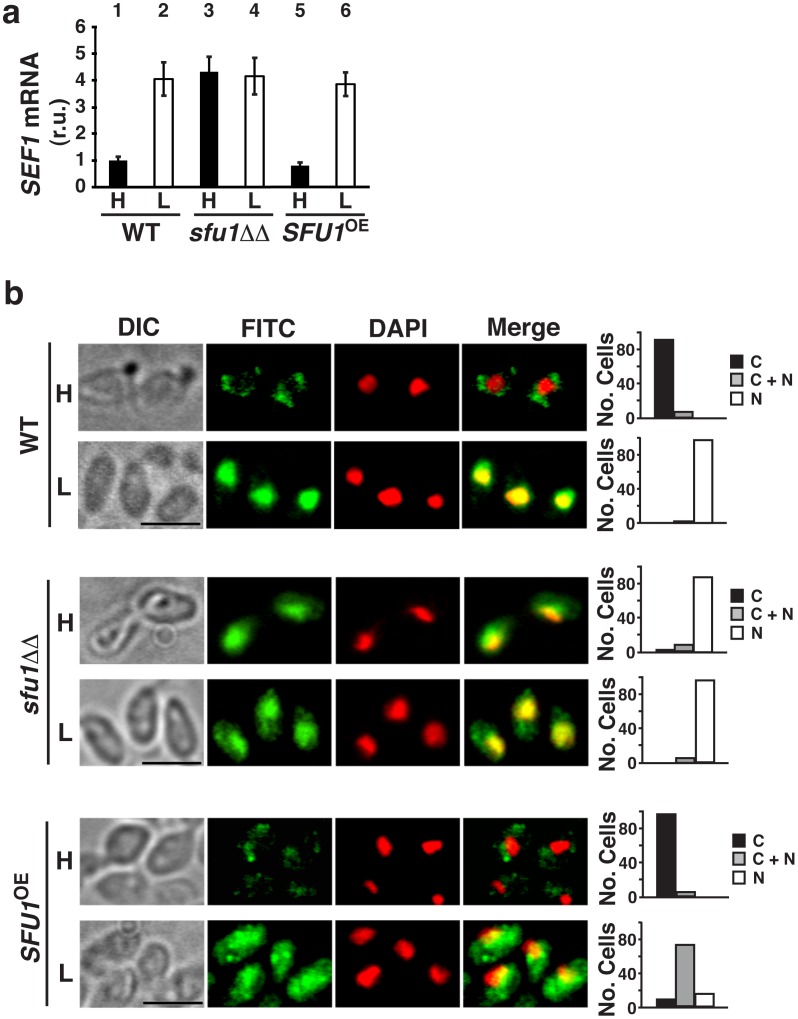
Sfu1 regulates *SEF1* gene expression and localization of Sef1 protein. Note that *C. albicans* gene deletion mutants are represented as *ΔΔ* because two alleles of target genes must be disrupted in this obligate diploid species. a) RT-qPCR results for *SEF1* mRNA in wild-type (WT), *sfu1ΔΔ*, and *SFU1-*overexpression (*SFU1* ^OE^) strains grown under iron-replete (H, high iron) or iron-depleted (L, low iron) conditions. Error bars correspond to the standard deviation among quintuplicate (H) or septuplicate (L) biological samples. Numerical values and statistical analysis are provided in **Table S1**). b) Indirect immunofluorescence of Sef1-Myc in WT, *sfu1ΔΔ*, and *SFU1* ^OE^ strains grown in iron-replete or iron-depleted medium. DIC represents phase images, FITC represents Sef1-Myc staining, DAPI represents DNA staining, and Merge represents the overlay of Sef1-Myc and DNA staining. Quantification of 100 cells in each experiment is shown on the right, with C representing >90% cytoplasmic staining, N >90% nuclear staining, and C+N a mixture of cytoplasmic and nuclear staining. Scale bar, 5 µm; all images were obtained at the same magnification.

Given the important role of Sef1 in promoting *C. albicans* virulence [6], we speculated that it would be a prime target for regulation. We and others had previously shown that, under iron-replete conditions, transcription of *SEF1* is repressed by Sfu1 [6], [10], the *C. albicans* structural and functional ortholog of *S. pombe* Fep1 [22]. To determine whether additional regulators contribute to *SEF1* gene expression, we used RT-qPCR to compare *SEF1* transcript levels in a wild-type strain vs. an isogenic strain lacking the *SFU1* gene. The result was that deletion of *SFU1* was sufficient to fully derepress *SEF1*, independent of the iron content of the growth medium (**Figure 1a**, compare the level of *SEF1* in wild-type cells grown in iron-depleted medium [bar 2, derepressing condition] to that in the *sfu1ΔΔ* strain, grown in either iron-replete [bar 3] or iron-depleted medium [bar 4]); numerical values and statistical analysis are provided in **Table S1**. These results suggested that iron-dependent transcriptional repression by Sfu1 is sufficient to account for *SEF1* transcript levels in wild-type cells.

To determine whether forced overexpression of *SFU1* could further suppress *SEF1* gene expression, we created a strain in which the endogenous promoter of *SFU1* was replaced with the strong, constitutively active *TDH3* promoter (*SFU1* ^OE^); increased levels of *SFU1* RNA and protein were confirmed by RT-qPCR and immunoblot analysis, respectively (**Figure S1a and S1b**). Overexpressed Sfu1 did not substantially diminish the level of *SEF1* mRNA under iron-replete or iron-depleted conditions (**Figure 1a**, compare bar 1 with bar 5 and bar 2 with bar 6). The failure of overexpressed *SFU1* to inhibit the transcription of *SEF1* under iron-depleted conditions suggested that *C. albicans* Sfu1 might, like its *S. pombe* ortholog [18], require iron as a cofactor for binding to DNA and transcriptional repression.

### Sfu1 promotes Sef1 localization in the cytoplasm

Although *SEF1* mRNA levels were normal in the *SFU1*-overexpression strain (**Figure 1a**), this strain demonstrated hypersensitivity to treatment with the iron chelator, bathophenanthroline disulfonic acid (BPS; **Figure S2**), suggestive of a potential defect in iron acquisition. Addition of FeCl_3_ to the BPS-treated medium was sufficient to reverse the growth defect (**Figure S2**), confirming the specificity of the iron-chelation phenotype. To determine whether Sef1 protein levels were affected in the *SFU1* ^OE^ strain, we utilized an epitope-tagged version of Sef1 in which 13 copies of the Myc epitope were fused in-frame at the C-terminus; this fusion protein is fully functional [6]. Surprisingly, the steady state level of Sef1-Myc was substantially reduced, particularly under iron-depleted conditions (**Figure S3**). The observations that overexpression of *SFU1* does not affect *SEF1* mRNA levels but strongly decreases Sef1 protein levels raised the possibility that Sfu1 may have a second function in the post-transcriptional regulation of Sef1.

To determine whether Sef1 localization is regulated, we used indirect immunofluorescence to visualize Sef1-Myc in wild-type cells exposed to varying concentrations of iron. Under iron-replete conditions, Sef1-Myc was localized primarily in the cytoplasm (**Figure 1b**, WT strain, H; note the absence of green Sef1-Myc signal in the FITC channel in areas that correspond to red DNA signal in the DAPI channel; a negative control showing minimal staining of an isogenic strain that lacks the Myc epitope is shown in **Figure S4a**). Under iron-depleted conditions, however, Sef1-Myc was primarily nuclear, with prominent areas of yellow overlap when the FITC and DAPI channels were merged. Notably, examination of Sef1-Myc in an *sfu1ΔΔ* mutant revealed constitutive nuclear localization, even under iron-replete conditions (*sfu1ΔΔ* strain, **Figure 1b**). Conversely, overexpression of *SFU1* resulted in substantial cytoplasmic localization of Sef1-Myc even under iron-depleted conditions in which it is usually nuclear (*SFU1* ^OE^ strain, **Figure 1b**). By comparison, an Sfu1-Myc fusion protein was found to be distributed between the nucleus and cytoplasm in wild-type cells propagated under iron-replete conditions and primarily cytoplasmic under iron-limiting conditions (**Figure S4b**).

These results established that Sef1 localization varies as a function of iron, that Sfu1 promotes Sef1 localization in the cytoplasm, and that the protein localizing activity of Sfu1—unlike its transcriptional repression activity (**Figure 1a**)—does not inherently require iron.

### Ssn3 promotes Sef1 phosphorylation and nuclear localization under iron-depleted conditions

**Figure 2:**
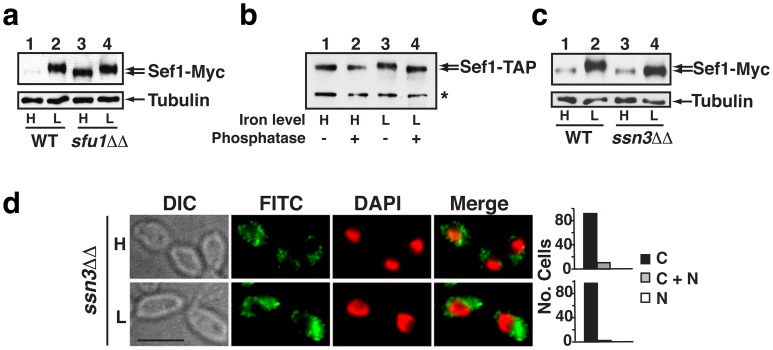
Ssn3 promotes phosphorylation and nuclear localization of Sef1 under iron-depleted conditions. a) Immunoblot of Sef1-Myc and alpha tubulin (internal standard) in wild-type vs. *sfu1ΔΔ* cells propagated under iron-replete (H) or iron-depleted (L) conditions. B) Immunoblot of purified Sef1-TAP protein either treated (+) or not treated (−) with lambda phosphatase. To accumulate sufficient quantities of the higher mobility form of Sef1-TAP (lanes 1 and 2), the protein was purified from an *sfu1ΔΔ* mutant grown under iron-replete conditions. The lower mobility form (lanes 3 and 4, prior to phosphatase treatment) was purified from wild-type cells grown under iron-depleted conditions. * indicates a presumed Sef1-Myc C-terminal proteolysis product that is observed only when Sef1 is recovered from nondenaturing extracts, such as those used for TAP purification; this fragment is not seen when denaturing extracts are used as in (a) above and (c) below. c) Immunoblot of Sef1-Myc and alpha tubulin recovered from wild-type or *ssn3ΔΔ* cells under iron-replete or iron-depleted conditions. d) Indirect immunofluorescence of Sef1-Myc in the *ssn3ΔΔ* mutant under iron-replete or iron-depleted conditions. Scale bar, 5 µm; all images obtained at the same magnification.

Immunoblot analysis of Sef1-Myc recovered from wild-type cells grown under iron-replete vs. iron-depleted conditions demonstrated an inverse relationship between Sef1 protein abundance and iron levels (**Figure 2a**, lanes 1 and 2), which was expected based on the known, iron-dependent inhibitory activity of Sfu1 on *SEF1* gene expression. An unexpected finding was that the electrophoretic mobility of Sef1 also varied in an iron-dependent fashion. This subtle but reproducible decrease in Sef1 mobility under iron-depleted conditions was observed not only in wild-type cells, but also in an *sfu1ΔΔ* deletion mutant (lanes 3 and 4), arguing against a role for Sfu1 in this process.

We hypothesized that the lower mobility form of Sef1 might result from covalent phosphorylation. To test this hypothesis, we used a tandem affinity purification strategy to recover TAP-tagged Sef1 from *C. albicans* grown under iron-replete or iron-depleted conditions. Purified TAP-tagged Sef1 exhibited an iron-dependent mobility shift similar to that of Sef1-Myc, with protein from the iron-depleted cells running with slightly lower mobility (**Figure 2b**, compare lanes 1 and 3). Treatment of the purified proteins with lambda phosphatase, a broad specificity enzyme with activity on phospho-serine, phospho-threonine, and phospho-tyrosine residues, resulted in conversion of the lower mobility form of Sef1-TAP to the higher mobility form (**Figure 2b**, compare lane 4 to lanes 1 and 2), in support of our hypothesis.

To identify the kinase responsible for low-iron-dependent phosphorylation of Sef1, we tested the 31 available homozygous knockout mutants affecting predicted kinases for sensitivity to BPS. Our reasoning was that, if phosphorylation of Sef1 is required for full induction of iron uptake genes, then a mutant lacking the relevant kinase might be hypersensitive to iron depletion, that is, phenotypically similar to *sef1ΔΔ* itself [6], [23]. Our screen identified the *ssn3ΔΔ* mutant as being hypersensitive to iron depletion (**Figure S2**). Further, an immunoblot of Sef1-Myc recovered from the *ssn3ΔΔ* strain revealed persistence of the higher mobility form under iron-depleted conditions (**Figure 2c**), consistent with a role for Ssn3 in phosphorylation of Sef1. The identical result was obtained when Sef1-Myc was examined in a strain encoding a predicted kinase-dead allele of Ssn3 (Ssn3^D325A^, **Figure S5a**).

Although *C. albicans* Ssn3 has not yet been characterized, its *S. cerevisiae* ortholog is a cyclin-dependent kinase with two known functions: first, it is a component of the Mediator complex with inhibitory activity on RNA polymerase II [24]; second, it phosphorylates a number of specific transcription factors to regulate their activity, nuclear-cytoplasmic localization, and/or stability [25], [26], [27]. To determine whether *C. albicans* Ssn3 influences the localization of Sef1, we performed indirect immunofluorescence on Myc-tagged Sef1 in the *ssn3ΔΔ* mutant. As shown in **Figure 2d**, deletion of *SSN3* resulted in constitutive cytoplasmic localization of Sef1-Myc under both iron-replete and iron-depleted conditions; similar mislocalization was observed in a strain containing Ssn3^D325A^ (**Figure S5b**). Unlike the case with *SFU1*, however, overexpression of *SSN3* via the *TDH3* promoter (*SSN3* ^OE^) had no obvious effect on Sef1-Myc localization (**Figure S5b**), perhaps indicating that the nuclear localization activity of Ssn3 is restricted to low iron conditions.

### Sfu1 and Ssn3 each physically interact with Sef1 but play opposing roles

**Figure 3:**
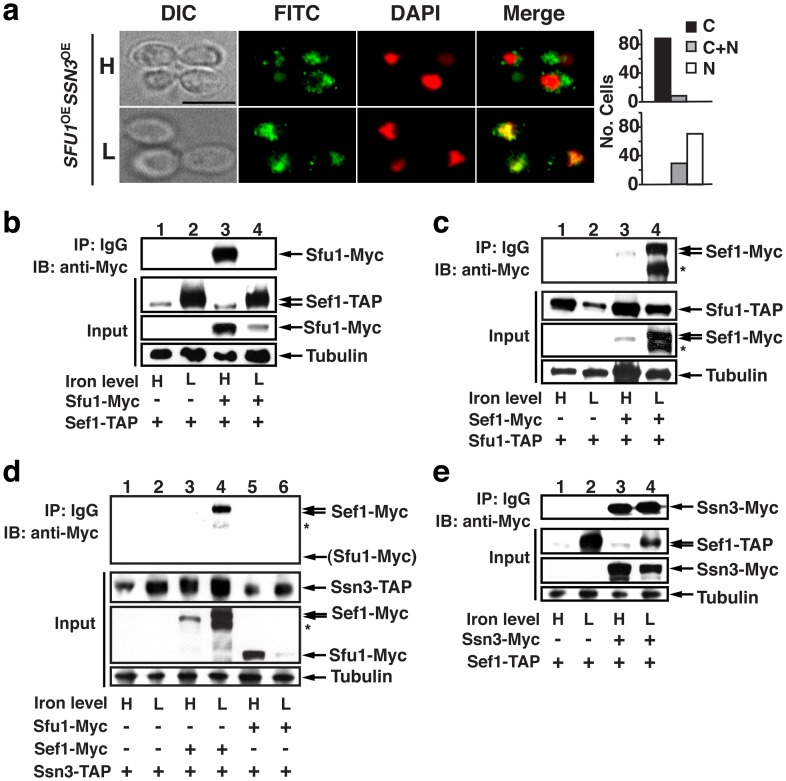
Sfu1 and Ssn3 each physically interact with Sef1 but play opposing roles in Sef1 localization. a) Overexpression of *SSN3* restores nuclear localization of Sef1 to a strain that also overexpresses *SFU1*. Indirect immunofluorescence of Sef1-Myc in a strain that constitutively overexpresses both *SFU1* and *SSN3*, shown for cells cultured in iron-replete (H) and iron-depleted (L) conditions. Scale bar, 5 µm; all images obtained at the same magnification. b) Sfu1-Myc is co-immunoprecipitated with Sef1-TAP. Wild-type strains containing only Sef1-TAP or both Sef1-TAP and Sfu1-Myc were grown under iron-replete or iron-depleted conditions. Whole cell extracts were prepared under nondenaturing conditions, and IgG-sepharose was used to immunoprecipitate Sef1-TAP and associated proteins. Pellets were subjected to immunoblot analysis, using anti-Myc monoclonal antibodies to identify Sfu1-Myc. c) Sef1-Myc is co-immunoprecipitated with Sfu1-TAP. * indicates a presumed Sef1-Myc C-terminal proteolysis product that is observed only when Sef1 is recovered from nondenaturing extracts, such as those used for TAP purification. d) Sef1-Myc but not Sfu1-Myc is co-immunoprecipitated with Ssn3-TAP. e) Ssn3-Myc is co-immunoprecipitated with Sef1-TAP. * Presumed Sef1-Myc C-terminal proteolysis product, see above.

The preceding results were suggestive of a model in which Sfu1 and Ssn3 have opposite and competing roles in Sef1 localization, with Sfu1 promoting cytoplasmic localization and Ssn3 promoting nuclear localization. To test this model, we utilized the *SFU1-*overexpression strain that mislocalizes Sef1-Myc to the cytoplasm under iron-depleted conditions (**Figure 1b**). We predicted that, if Ssn3 competes with Sfu1 for localization of Sef1, then overexpression of *SSN3* might rescue this Sef1 mislocalization phenotype. Indeed, a strain in which both genes are driven by the strong *TDH3* promoter exhibits substantial restoration of nuclear Sef1-Myc under iron-depleted conditions, with normal cytoplasmic localization under iron-replete conditions (**Figure 3a**). These results indicate that Sfu1 and Ssn3 exert opposing roles on Sef1 localization, but only under iron-depleted conditions (when Sef1 is phosphorylated).

To determine whether Sef1 physically associates with Sfu1 and/or Ssn3, we created a series of double epitope-tagged strains, each containing a Myc-tagged version of one of the three potentially interacting proteins and a TAP-tagged version of another; the TAP epitope consists of a calmodulin binding domain fused to a TEV cleavage site and a Protein A domain (**Figure S6a**; [28], [29]). Co-immunoprecipitation experiments were performed using whole cell extracts prepared from cells grown under iron-replete or iron-depleted conditions. Extracts were incubated with IgG-sepharose, which binds to the Protein A component of the TAP epitope, followed by extensive washing of the immunoprecipitated complexes and protein electrophoresis under denaturing conditions (SDS-PAGE; see **Figure S6b** for a schematic of the protocol). Finally, immunoblots were probed with anti-Myc antibodies to determine the presence or absence of a Myc-tagged putative binding partner. Specificity of IgG-sepharose for the TAP tag was confirmed by performing experiments with strains containing Myc-tagged fusion proteins and an unfused TAP tag (**Figure S6c**), and specificity of the anti-Myc antibodies for the Myc epitope was confirmed using cells containing only the TAP-tagged fusion proteins (**Figure 3b**).

Shown in **Figure 3b** are the results with Sfu1-Myc and Sef1-TAP. Sfu1-Myc was efficiently co-immunoprecipitated with Sef1-TAP when cells were propagated in iron-replete medium (lane 3, IP), but not when iron-starved cells were used (lane 4, IP). On the other hand, when the epitope tags were reversed, co-immunoprecipitated Sef1-Myc was poorly visualized using extracts of iron-replete cells (**Figure 3c**, lane 3) but was easily seen using iron-starved cells (**Figure 3c**, lane 4). Together, these results suggest that Sef1 and Sfu1 interact physically in a manner that is independent of iron levels, whereas the sensitivity of our biochemical assay is a function of the relative abundance of the Myc-tagged protein in the extract.

Co-immunoprecipitation experiments combining either Sef1-Myc or Sfu1-Myc with Ssn3-TAP revealed a robust interaction between Ssn3 and Sef1, but no detectable interaction between Ssn3 and Sfu1 (**Figure 3d**). That is, Sef1-Myc was efficiently co-immunoprecipitated with Ssn3-TAP from an extract of iron-depleted cells (**Figure 3d**, lane 4, IP), which express relatively high amounts of Sef1-Myc protein (**Figure 3d**, lane 4, input), whereas Sfu1-Myc was not co-immunoprecipitated under any condition (**Figure 3d**, lanes 5 and 6, respectively). When the epitope tags were reversed, Ssn3-Myc was efficiently co-immunoprecipitated with Sef1-TAP using either iron-replete (**Figure 3e**, lane 3) or iron-depleted (**Figure 3e**, lane 4) cells; note that Ssn3-Myc is relatively abundant under both conditions. These results suggest that Sef1 physically associates with Ssn3 as well as Sfu1, but these appear to represent alternative complexes since Ssn3 and Sfu1 do not associate with each other.

### Sef1 is likely destabilized in the cytoplasm

**Figure 4:**
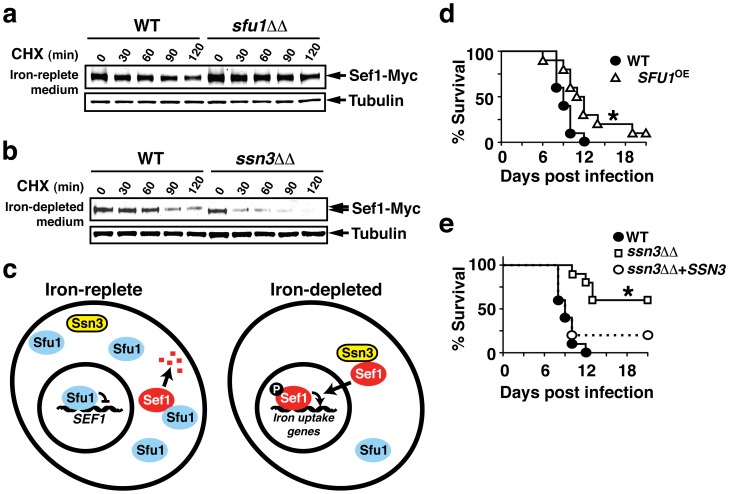
Sfu1 and Ssn3 mediate opposing effects on Sef1 stability and virulence. a) Sef1 protein is stabilized in an *sfu1ΔΔ* mutant. Wild-type or *sfu1ΔΔ* strains containing Sef1-Myc were propagated in iron-replete medium containing 2 mg/ml cycloheximide. Samples were recovered at the indicated time points, and Sef1-Myc was visualized using monoclonal antibodies against the Myc epitope, followed by incubation with secondary antibodies that were coupled to infrared dyes and quantified using a Li-Cor instrument. Note that a single band corresponding to higher mobility (unphosphorylated) Sef1 is present in both strains. b) Sef1 protein is destabilized in the *ssn3ΔΔ* mutant. The experiment was performed as above, except that cells were propagated in iron-depleted medium containing cycloheximide. Note that phosphorylated Sef1 recovered from wild-type cells runs with slower mobility. c) Model for Sef1 regulation by Sfu1 and Ssn3 under iron-replete vs. iron-depleted conditions. Note that, even under iron-replete conditions when nuclear Sfu1 functions as a transcriptional repressor, a cytoplasmic pool of Sfu1 is available (**Figure** **S4**) that could participate in Sef1 sequestration. d) Overexpression of *SFU1* leads to attenuated *C. albicans* virulence in a murine bloodstream infections model. * signifies p<0.02, log-rank test. E) Deletion of *SSN3* leads to attenuated *C. albicans* virulence, and restoration of one copy of wild-type *SSN3* complements the defect. * signifies p<0.0001.

To learn whether the stability of Sef1 varies with its intracellular localization, we determined the half-life of Myc-tagged Sef1 in wild-type *C. albicans* and in mutants in which Sef1 is stably localized in either the nucleus or the cytoplasm. Under iron-replete conditions, Sef1 is predominantly cytoplasmic in wild-type *C. albicans* but is mislocalized to the nucleus in *sfu1ΔΔ* (**Figure 1b**). To obtain sufficient Sef1 protein for the analysis and to uncouple the role of Sfu1 in Sef1 localization from its effects on *SEF1* transcription, we replaced the endogenous *SEF1* promoter with a constitutively active *TDH3* promoter in both wild-type and *sfu1ΔΔ* strains; overexpressed Sef1-Myc exhibited the same pattern of iron-dependent nuclear vs. cytoplasmic localization as Sef1-Myc expressed from its endogenous promoter (**Figure S7**). The strains were propagated to mid log phase in iron-replete medium, followed by addition of cycloheximide to block further translation, and serial sampling for measurements Sef1-Myc abundance. Shown in **Figure 4a** is a quantitative immunoblot of Sef1-Myc and alpha tubulin, which was used as an internal control for protein loading. Under these iron-replete conditions, the calculated half-life of cytoplasmic Sef1-Myc was ∼80 minutes (wild type, R^2^ = 0.94) and that of nuclear Sef1-Myc was ∼160 minutes (*sfu1ΔΔ*, R^2^ = 0.92). Next, we examined Sef1-Myc stability under iron-depleted conditions, in which the protein is predominantly nuclear in wild-type cells (**Figure 1b**) but mislocalized to the cytoplasm in the *ssn3ΔΔ* mutant (**Figure 2d**). Wild-type and *ssn3ΔΔ* strains expressing *SEF1-MYC* from the endogenous *SEF1* promoter were propagated in iron-depleted medium to mid-log phase, then treated with cycloheximide and visualized as above (**Figure 4b**). Under these iron-depleted conditions, the calculated half-life of nuclear Sef1-Myc (∼150 minutes in wild type; R^2^ = 0.98) was once again more stable than that of cytoplasmic Sef1-Myc (∼40 minutes in *ssn3ΔΔ*; R^2^ = 0.96). The most parsimonious explanation for these results is that Sef1 is degraded more rapidly in the cytoplasm than in the nucleus; however, we cannot exclude the possibility that Ssn3 and Sfu1 exert independent effects on Sef1 degradation that are unrelated to its intracellular localization.

Our current model of Sef1 regulation, which integrates these results with previously published findings [6], [10], [22], is depicted in **Figure 4c**. According to the model, Sef1 is subject to two distinct forms of Sfu1-mediated repression when environmental iron is replete: 1) transcriptional repression of the *SEF1* gene, through direct binding and repression of transcriptional initiation; and 2) post-translational inhibition of Sef1 protein, through direct binding and retention in the cytoplasm, where Sef1 is more rapidly degraded. Alternatively, under iron-limiting conditions, when Sfu1 protein is depleted, Sef1 is bound by Ssn3, phosphorylated, and localized in the nucleus, where it activates expression of iron uptake genes. Our recent observation that Sef1-Myc is constitutively cytoplasmic in an *sfu1ΔΔ/ssn3ΔΔ* double mutant strain (**Figure S8**) suggests that Ssn3 may play actively promote the nuclear localization of Sef1, beyond merely extricating Sef1 from Sfu1.

### Nuclear localization of Sef1 is associated with virulence

We previously demonstrated that *SEF1* gene expression is induced in the iron-limiting environment of the host bloodstream and that *SEF1* is required for virulence in a murine model of bloodstream candidiasis [6]. Conversely, we showed that *SFU1* is not required for virulence but rather that the *sfu1ΔΔ* mutant exhibits increased competitive fitness relative to wild-type *C. albicans*, presumably because of an enhanced ability to take up extracellular iron [6]. Our current results suggest that the negative effect of Sfu1 on *C. albicans* virulence likely results from mislocalization of Sef1 to the cytoplasm rather than from repression of *SEF1* gene expression, since only the former activity is observed under conditions of iron depletion (compare **Figure 1a** and **Figure 1b**). We tested this hypothesis by examining the virulence of mutants with moderate (*SFU1* ^OE^, **Figure 1b**, low iron condition) to severe (*ssn3ΔΔ*, **Figure 2d**, low iron condition) defects in Sef1 nuclear localization. As shown in **Figure 4d and 4e**, both mutants were significantly attenuated in the murine bloodstream infection model, such that mice infected with either mutant survived longer than mice infected with wild type. Note also that the strength of the virulence defects paralleled the strength of the Sef1 mislocalization defects of the two mutants, with those of *ssn3ΔΔ* being worse, although contributions from additional misregulated targets of Ssn3 cannot be excluded.

## Discussion

Human pathogenic microorganisms encounter a dearth of iron in the host bloodstream and internal organs [30], [31], [32], and specialized systems for iron acquisition have been demonstrated to be essential for the virulence of numerous bacterial, fungal, and parasitic pathogens, e.g. [33], [34], [35], [36], [37], [38], [39], [40]. Meanwhile, commensal-pathogens such as *C. albicans* face the additional challenge of potential iron toxicity (from free radicals generated by the Fenton reaction [41]) in niches such as the gastrointestinal tract, where iron is relatively abundant [8], [9]. We previously showed that the *C. albicans* transcription factors Sef1 and Sfu1 are key components of an iron homeostasis regulatory circuit that permits adaptation to these widely divergent host niches [6]. Sef1 protects against iron deficiency in the bloodstream through the induction of iron uptake genes and repression of *SFU1*, whereas Sfu1 protects against iron toxicity in the gut through repression of iron uptake genes as well as *SEF1*. Still unanswered are the questions of how the activities of Sef1 and Sfu1 are themselves tied to iron levels and whether additional regulatory inputs are involved. Here, we define a system for post-transcriptional, iron-dependent regulation of Sef1 protein that precisely controls the virulence of this obligate commensal-pathogen.

### A novel post-transcriptional regulatory mechanism controls iron homeostasis in *C. albicans*

Sef1 plays a central role in *C. albicans* pathogenesis through promoting the expression of virulence factors as well as iron uptake genes, whereas Sfu1 is essential for commensalism [6]. Given its role in virulence and, perhaps, in the choice between commensal and virulent lifestyles, we hypothesized that Sef1 would be a prime target for regulation beyond transcriptional repression by Sfu1. Indeed, our analysis of Myc-tagged Sef1 in wild-type *C. albicans* has revealed multiple levels of iron-dependent regulation, including nuclear vs. cytoplasmic localization, phosphorylation, and differential protein stability. In wild-type cells, Sef1 protein is nuclear, phosphorylated, stable, and competent for transcriptional activation only under iron-depleted conditions such as those encountered in the bloodstream.

Our analysis of Sef1 in *C. albicans* mutants has shed further light on the mechanisms of Sef1 regulation. Surprisingly, in the *sfu1ΔΔ* mutant, Sef1-Myc is constitutively nuclear, whereas in an *SFU1-*overexpression strain it is predominantly cytoplasmic. These results clearly suggested a role for Sfu1 in the cytoplasmic localization of Sef1. Our screen of *C. albicans* mutants affecting predicted kinases exposed a role for Ssn3 in promoting cellular resistance to iron depletion as well as phosphorylation of Sef1. Co-immunoprecipitation experiments indicating that Ssn3 forms a physical complex with Sef1 supported a direct role for Ssn3 in Sef1 phosphorylation. Our finding that Sef1-Myc is constitutively cytoplasmic in the *ssn3ΔΔ* mutant suggested that Ssn3 might oppose Sfu1 by promoting the nuclear localization of Sef1. This hypothesis was validated by the ability of overexpressed *SSN3* to overcome the cytoplasmic Sef1-mislocalization phenotype (under low iron conditions) of an *SFU1-*overexpression strain. Finally, our observations that Sfu1 and Ssn3 were both detectable in complexes with Sef1, but that neither could be found associated with the other, suggested that the functional antagonism between Sfu1 and Ssn3 occurs in part through competitive binding to Sef1 protein. Meanwhile, the observation that Sef1 is constitutively cytoplasmic in an *sfu1ΔΔ/ssn3ΔΔ* double mutant argues that Ssn3 plays at least one additional role in Sef1 nuclear localization.

These studies led to a revised model of Sef1 regulation (**Figure 4c**). According to the model, under iron-replete conditions, Sfu1 utilizes two distinct mechanisms to inhibit the function of Sef1: 1) Transcriptional repression, via direct binding to the *SEF1* promoter, and 2) Post-transcriptional repression, via binding to Sef1 protein and forced localization in the cytoplasm, where Sef1 is unstable and unable to participate in transcription. To our knowledge, this would be the first example of a regulatory factor that regulates it target by both transcriptional and post-transcriptional mechanisms. Under iron-limiting conditions, Sfu1 protein is depleted, and Sef1 associates with the predicted protein kinase, Ssn3. Ssn3 most likely phosphorylates Sef1 directly, and either the complex or free Sef1 is transported to the nucleus, where Sef1 functions as a transcriptional activator. A key goal of future studies will be to understand how iron regulates these newly described activities of Sfu1 and Ssn3.

### Impact of Sef1 post-transcriptional regulation on virulence

The findings that Ssn3 and Sfu1 post-transcriptionally regulate Sef1, an important virulence factor, raised the question of whether these regulatory events impact *C. albicans* virulence. Previously, we observed that deletion of *SFU1* leads to hypervirulence in the murine bloodstream infection model, with the *sfu1ΔΔ* mutant significantly better at colonizing host kidneys than wild-type *C. albicans* [6]. We attributed this enhanced fitness to derepression of *SEF1* and iron uptake genes in the mutant, resulting in an increased capacity for iron acquisition. In light of our current results showing that Sfu1 requires iron for transcriptional repression activity, a more likely explanation for the fitness advantage of *sfu1ΔΔ* is that Sef1 is constitutively nuclear (and therefore transcriptionally active) in this strain, whereas in wild type some fraction of Sef1 is retained in the cytoplasm and degraded. Our current observations with *SFU1* ^OE^ and *ssn3ΔΔ* mutants dovetail with these findings by showing the converse, i.e. that mutants with incremental defects in the nuclear localization of Sef1 have proportional defects in virulence. Together, these results strongly support the hypothesis that *C. albicans* iron acquisition (and therefore virulence) can be modulated up or down, respectively, through the activities of Ssn3 or Sfu1 on Sef1 localization and stability. We hypothesize that the evolution of such fine-tuned regulation of a potent transcription factor is particularly advantageous to an obligate commensal-pathogen, such as *C. albicans*, which must continuously adapt to differing iron concentrations among the various microenvironments of its mammalian host, while avoiding excessive expression of pathogenicity genes during its usual role as a commensal.

## Materials and Methods

### Ethics statement

All procedures involving animals were approved by the Institutional Animal Care and Use Committee at the University of California San Francisco and were carried out according to the National Institutes of Health (NIH) guidelines for the ethical treatment of animals.

### Media

*C. albicans* strains were routinely propagated in YPD, also referred to as “iron-replete” medium. “Iron-depleted” medium is YPD supplemented with one of the specific iron chelators, bathophenanthroline disulfonic acid (BPS) or 2,2′-dipyridyl (DIP), as previously described [6].

### Plasmid and strain construction

All *C. albicans* strains used in this study are described in **Table S2**, primers are listed in **Table S3**, and plasmids are listed in **Table S4**. Construction of *C. albicans* knockout mutants, complemented (gene addback) strains, and strains containing Myc-tagged fusion proteins was performed as previously described [6], [42], [43], [44].

For introduction of TAP epitopes at the C-terminus of Sef1, Sfu1, and Ssn3, a series of plasmids was constructed using PCR and homologous recombination in *S. cerevisiae* [45]. The vector was pRS316 [46], and the insert consisted of (5′ to 3′): a PmeI restriction site; 350–450 bp of target ORF sequence up to, but not including, the stop codon; the TAP tag coding sequence [29]; a *SAT1* (dominant selectable marker)*-*flipper cassette [47]; 350–450 bp of sequence downstream of the target ORF; and a second PmeI restriction site. Plasmids were called pSN150 (Sef1-TAP), pSN228 (Sfu1-TAP), and pSN219 (Ssn3-TAP). PmeI-digested plasmids were transformed into wild-type *C. albicans* reference strain SN250 [42], and nourseothricin-resistant *C. albicans* transformants were screened by colony PCR to verify the correct orientation of the C-terminal TAP tag and *SAT1*-flipper cassette. Strains expressing both Myc- and TAP-tagged fusion proteins were constructed by transforming strains already expressing the Myc-tagged protein with the appropriate PmeI-digested TAP-tag integration fragment, as described above.

Overexpression strains for *SEF1-* and *SSN3* were created by replacing portions of the endogenous promoters with the highly active *TDH3* promoter. PCR and homologous recombination in *S. cerevisiae* [45] were used to create plasmids containing (5′ to 3′): a PmeI restriction site; 350–450 bp of sequence homology ending ∼500 bp upstream of the target ORF; the *SAT1* gene (dominant selectable marker); the *TDH3* promoter; 350–450 bp of sequence homology beginning with the start codon of the target ORF; and a second PmeI site. The vector was pRS316 [46], the source of *NAT1-TDH3* promoter was pCJN542 [48], and the resulting plasmids were named pSN147 (*SEF1* ^OE^) and pSN229 (*SSN3* ^OE^). Correct integration of the inserts in nourseothricin-resistant transformants was verified by colony PCR, and overexpression of *SEF1* and *SSN3* was confirmed by RT-qPCR.

The *SFU1* overexpression strain (SN742) was created using an analogous method. pSN141 was engineered to contain (5′ to 3′): a PmeI site; 350–450 bp of sequence upstream of the *C. albicans LEU2* ORF; the *C. dubliniensis ARG4* gene (selectable marker); the *TDH3* promoter; the *SFU1* ORF; 350–450 bp sequence downstream of the *LEU2* ORF; and a second PmeI restriction site. After digestion with PmeI, the plasmid was transformed into SN515 (*sfu1ΔΔ*). Correct integration of the insert in Arg^+^ transformants was verified by colony PCR, and overexpression of *SFU1* was confirmed by RT-qPCR.

The Ssn3^D325A^ kinase-dead mutant (SN977) was created in a similar manner to that of the *SFU1* ^OE^ strain. First, PCR and primers SNO1394 through SNO1397 (**Table S3**) were used to create a D325A-encoding variant of the *SSN3* ORF. Next, plasmid pSN239 was engineered to contain (5′ to 3′): a PmeI site; 350–450 bp of sequence upstream of the *C. albicans LEU2* ORF; *C. dubliniensis ARG4* (selectable marker); the *TDH3* promoter; the mutant *SSN3* ORF; 350–450 bp sequence downstream of the *LEU2* ORF; and a second PmeI restriction site. A Myc-tagged version of Ssn3^D325A^ (SN987) was created using a plasmid (pSN273) that contains (5′ to 3′): a PmeI site; 350–450 bp of *SSN3* ORF sequence up to, but not including, the stop codon; sequence encoding 13×Myc; a *SAT1-*flipper cassette [47]; 272 bp of sequence downstream of the *SSN3* ORF; 350–450 bp of sequence downstream of the *LEU2* ORF; and a second PmeI restriction site. PmeI-digested plasmid was transformed into SN977, and correct integration in nourseothricin-resistant transformants was verified by colony PCR. Sequences of all PCR products were verified by DNA sequencing.

### Fluorescence microscopy

*C. albicans* was grown at 30°C for 5–6 hours in “iron-replete” (YPD) or “iron-depleted” medium (YPD supplemented with 500 µM BPS) to OD_600_ = 0.8–1.0. Cell fixation, cell wall digestion, and antibody hybridization were performed as previously described [49] except that the 9E10 anti-c-Myc antibody (Covance Research) was used at a 1∶300 dilution and detected with a 1∶400 dilution of Cy2-conjugated secondary antibody (Jackson ImmunoResearch, 715-225-151). Images were acquired under 100× oil objective using a cooled CCD camera (Cooke Sensicam) mounted on an inverted microscope (Zeiss Axioplan 200 M; Carl Zeiss MicroImaging) or a Nikon Eclipse TE2000-E fluorescence microscope. All images were processed with ImageJ software (National Institutes of Health).

### Protein extraction and immunoblotting

*C. albicans* protein extracts were prepared under denaturing condition using a protocol adapted from a previously described method [50]. Lysates corresponding to 1 OD_600_ of cells were analyzed by SDS-PAGE and immunoblotted with either anti-c-Myc (9E10, Covance Research) for Myc-tagged proteins or anti-peroxidase soluble complex antibody (Sigma, P2416) for TAP-tagged proteins. Immunoblots were also probed with anti-alpha tubulin antibody (Novus Biologicals, NB100-1639) as a loading control.

### Quantification of Sef1 half-life

*C. albicans* strains were grown on YPD medium (“iron-replete”) or YPD medium supplemented with the specific iron chelator 2,2′-dipyridyl (DIP) at a final concentration of 0.5 mM (“iron-depleted”). A sample of 1–1.5 OD_600_ cells was taken immediately (zero time point) before addition of cycloheximide to a final concentration of 2 mg/ml. At the indicated times, 1 OD value of cells was collected and harvested for protein preparations and immunoblotting. Semiquantitative detection of protein levels was performed using the LiCor Odyssey Infrared Imager (Lincoln, NE). Integrated fluorescence intensities of individual bands were measured and background subtracted using the Odyssey Application software. The signal from Sef1-Myc bands was normalized to that of alpha tubulin. Calculations of half-life were performed as previously described [51].

### TAP pull-down analysis

Cells expressing TAP-tagged Sef1, Sfu1 or Ssn3 were grown in YPD medium to OD_600_ = ∼0.3–0.35 and centrifuged for 5 min at 3,000 rpm. Cell pellets were resuspended in “iron-replete” medium (pre-warmed YPD) or “low iron” medium (pre-warmed YPD supplemented with DIP), and grown for an additional 4 hours in the dark. Cells were collected by centrifugation, washed three times with ice-old water, and resuspended in 1 ml of lysis buffer (20 mM Tris, pH 7.4, 100 mM KCl, 5 mM MgCl_2_, 20% glycerol) with protease and phosphatase inhibitors (Roche). Cells were lysed using a Bead Beater and one-third volume of glass beads. Cell lysates were centrifuged for 2×20 minutes at 14,000 rpm at 4°C. Protein concentration of the supernatants was measured by the Bradford assay. 3 mg of proteins was used for immunoprecipitation with 50 µl of immunoglobulin G-Sepharose resin (IgG Sepharose 6 Fast Flow, GE Healthcare). After 24 h of protein binding with rotation at 4°C, the resin was washed 4 times with lysis buffer and 2 times with tobacco etch virus (TEV) protease cleavage buffer (10 mM Tris-HCl, pH 8, 150 mM NaCl, 0.5 mM EDTA, 0.1% Tween-20). TEV protease (100 U) cleavage was performed in 1 ml buffer at 4°C overnight. The TEV eluate was collected and proteins were recovered by TCA (trichloroacetic acid) precipitation.

### RNA extraction and RT-qPCR analyses

Total RNA was prepared using a hot-phenol method [52] and treated with DNaseI using the Turbo DNA-free kit (Ambion). Ten micrograms of RNA was used in standard RT reactions using oligo [(dT)20-N] primers. cDNAs were quantified by qPCR with the primers listed in **Table S3** and normalized against *ACT1*.

### Virulence analysis

As previously described [6], groups of 10 female (8- to 10-week-old) BALB/c mice (Charles Rivers) were injected by tail vein with 5×10^5^ CFUs of wild type (SN425), *SFU1* ^OE^ (SN742), *ssn3ΔΔ* (SN982), or *ssn3ΔΔ*/SSN3 (SN978). Mice were monitored twice daily and euthanized when morbidity criteria were met (weight loss >15%, hunched posture, inactivity).

## Supporting Information

Figure S1 **Fusion to** ***TDH3*** **promoter results in overexpression of** ***SFU1*** **RNA and protein.** Results are shown for strains propagated under iron-replete conditions. a) RT-qPCR analysis of *SFU1* RNA in wild-type and *SFU1* ^OE^ strains. b) Immunoblot of Sfu1-Myc protein in wild-type and *SFU1* ^OE^ strains.
(EPS)

Figure S2 **BPS-sensitivity of** ***C. albicans*** **mutants.** Dilutions of equivalent numbers of wild-type and mutant cells were plated on iron-replete medium (YPD), iron-depleted medium (YPD +300 µM BPS), and iron-depleted medium with supplemental iron (YPD +300 µM BPS +100 µM FeCl_3_) and incubated at 30°C. Note that the BPS-related growth defects of *SFU1^OE^, sef1ΔΔ, and ssn3ΔΔ* were complemented by FeCl_3_ supplementation, indicating that the phenotypes resulted from iron limitation (vs. chelation of an alternative divalent cation).
(EPS)

Figure S3 **Immunoblot of Sef1-Myc and alpha tubulin in wild type vs.** ***SFU1*** **^OE^.** Cells were propagated in iron-replete (H) or iron-depleted (L) liquid medium, and proteins from cell equivalents were visualized in lanes 1, 2, 4, and 5. To better visualize Sef1-Myc extracted from cells grown under iron-depleted conditions, proteins from ¼ cell equivalents were visualized in lanes 3 and 6.
(EPS)

Figure S4 **Specificity of anti-Myc antibody for indirect immunofluorescence and localization of Sfu1-Myc.** a) Shown is indirect immunofluorescence of a *C. albicans* control strain that lacks a Myc epitope (SN250), showing minimal nonspecific staining with the anti-Myc antibody. b) Shown are indirect immunofluorescence images of an isogenic strain containing *SFU1-MYC*. H and L denote iron-replete vs. iron-depleted conditions, respectively, and results for 100 cells in each condition are graphed on the right.
(EPS)

Figure S5 **Ssn3 promotes covalent modification and nuclear localization of Sef1.** a) Immunoblot of Sef1-Myc and alpha tubulin in wild-type, *ssn3ΔΔ*, and *SSN3^OE^*cells propagated under iron-replete (H) and iron-depleted (L) conditions. b) Indirect immunofluorescence of Sef1-Myc in strains that contain a predicted kinase-dead allele of Ssn3 (*ssn3^D325A^*) or that overexpress the wild-type allele of *SSN3* (*SSN3^OE^*).
(EPS)

Figure S6 **Specificity of IgG-sepharose immunoprecipitation of the TAP tag.** a) Cartoon of a TAP-tagged fusion protein. b) Schematic of the IgG-sepharose co-immunoprecipitation protocol. c) Results of co-immunoprecipitation experiments using cells that overexpress an unfused TAP peptide along with Sef1-Myc, Sfu1-Myc, or Ssn3-Myc. Cells were propagated under conditions that would maximize the abundance of the Myc-tagged proteins (iron-depleted for Sef1-Myc, iron-replete for Sfu1-Myc and Ssn3-Myc), which were visualized using the anti-Myc antibody. The TAP peptide was visualized using an antibody (anti-PAP) that recognizes a portion of Protein A. Input, Flow Through (FT), and Eluate designations correspond to fractions depicted in (b). Note that TAP peptides are not visible in Eluate fractions because cleavage with TEV protease liberates only the CBP portion of TAP, whereas the Protein A portion remains bound to IgG-sepharose.
(EPS)

Figure S7 **Localization of overexpressed Sef1-Myc.** Shown are indirect immunofluorescence images of a strain containing *SEF1-MYC* under control of the *TDH3* promoter. H and L denote iron-replete vs. iron-depleted conditions, respectively, and results for 100 cells in each condition are graphed on the right.
(EPS)

Figure S8 **Sef1-Myc is constitutively cytoplasmic in a** ***sfu1ΔΔ/ssn3ΔΔ*** **double mutant strain.** Shown are indirect immunofluorescence images of Sef1-Myc in an *sfu1ΔΔ/ssn3ΔΔ* strain, with H and L denoting iron-replete vs. iron-depleted conditions, respectively. On the right are results for 100 cells in each condition.
(EPS)

Table S1 **Quantitation and statistical analysis of** ***SEF1*** **mRNA levels in various strains.**
(DOCX)

Table S2 **Strains used in this study.**
(DOCX)

Table S3 **Primers used in this study.**
(DOCX)

Table S4 **Plasmids used in this study.**
(DOCX)
