# Supplementary material for: Post-Transcriptional Regulation of the Sef1 Transcription Factor Controls the Virulence of Candida albicans in Its Mammalian Host
Source: PLoS Pathog. 2012 Nov 1;8(11):e1002956. doi: 10.1371/journal.ppat.1002956 (PMC3486892; doi:10.1371/journal.ppat.1002956)
Supplement: Table S1 — Quantitation and statistical analysis of SEF1 mRNA levels in various strains. (DOCX) [file ppat.1002956.s009.docx]

**Table S1. (a) Quantitation and (b) statistical analysis of *SEF1* RT-qPCR results in various strains**

**a**

|  | ***SEF1* level (relative units)** | |
| --- | --- | --- |
| **Strain-Iron level** | **Median** | **Standard deviation** |
| **WT-H** | **1.000** | **0.133** |
| **WT-L** | **4.048** | **0.623** |
| ***sfu1∆∆* -H** | **4.322** | **0.557** |
| ***sfu1∆∆*-L** | **4.154** | **0.686** |
| ***SFU1*OE-H** | **0.805** | **0.113** |
| ***SFU1*OE-L** | **3.856** | **0.441** |

**b**

| **Significance of difference between *SEF1* levels in pairs of strains** | | |
| --- | --- | --- |
| **Comparitor 1** | **Comparitor 2** | **p value** |
| **WT-H** | **WT-L** | **0.0012 *** |
| **WT-H** | ***sfu1ΔΔ*-H** | **0.0002 *** |
| **WT-H** | ***sfu1ΔΔ*-L** | **0.0004 *** |
| **WT-H** | ***SFU1*OE-H** | **0.0009 *** |
| **WT-H** | ***SFU*1OE-L** | **0.0001 *** |
| **WT-L** | ***sfu1ΔΔ*-H** | **0.4114** |
| **WT-L** | ***sfu1ΔΔ*-L** | **0.8937** |
| **WT-L** | ***SFU1*OE-H** | **0.0005 *** |
| **WT-L** | ***SFU*1OE-L** | **0.0922** |
| ***sfu1∆∆*-H** | ***sfu1ΔΔ*-L** | **0.837** |
| ***sfu1∆∆*-H** | ***SFU1*OE-H** | **0.0001 *** |
| ***sfu1∆∆*-H** | ***SFU*1OE-L** | **0.0108 *** |
| ***sfu1∆∆*-L** | ***SFU1*OE-H** | **0.0003 *** |
| ***sfu1∆∆*-L** | ***SFU*1OE-L** | **0.2876** |
| ***SFU1*OE-H** | ***SFU*1OE-L** | **< 0.0001 *** |

* Significant difference, defined as p-value < 0.05 by the t-test
