# Supplementary material for: Post-Transcriptional Regulation of the Sef1 Transcription Factor Controls the Virulence of Candida albicans in Its Mammalian Host
Source: PLoS Pathog. 2012 Nov 1;8(11):e1002956. doi: 10.1371/journal.ppat.1002956 (PMC3486892; doi:10.1371/journal.ppat.1002956)
Supplement: Table S2 — Strains used in this study. (DOCX) [file ppat.1002956.s010.docx]

**Table S2. Strains Used in this Study**

| **Strain** | **Relevant Genotype** | **Full Genotype** | **Reference** |
| --- | --- | --- | --- |
| SN250 | Wild type | *leu2Δ::C.m.LEU2/leu2Δ::C.d.HIS1, his1Δ/his1Δ, arg4Δ/arg4Δ, leu2Δ/leu2Δ ura3Δ/URA3, iro1Δ/IRO1* | Noble et al., 2010 |
| SN425 | Wild type (prototroph) | *leu2Δ::C.d.HIS1/ leu2Δ::C.m.LEU2, arg4Δ/arg4Δ::C.d.ARG4, his1Δ/his1Δ, ura3Δ/URA3, iro1Δ/IRO1* | Noble et al., 2010 |
| SN330 | *sef1****∆∆*** | *sef1 Δ::C.m.LEU2/sef1Δ::C.d.HIS1, his1Δ/his1Δ, arg4Δ/arg4Δ, leu2Δ/leu2Δ, ura3Δ/URA3, iro1Δ/IRO1* | Noble et al., 2010 |
| SN515 | *sfu1****∆∆*** | *sfu1Δ::C.m.LEU2/sfu1Δ::C.d.HIS1, his1Δ/his1Δ, arg4Δ/arg4Δ, leu2Δ/leu2Δ, ura3Δ/URA3, iro1Δ/IRO1* | Noble et al., 2010 |
| SN913 | *ssn3****∆∆*** | *ssn3Δ::C.m.LEU2/ssn3Δ::C.d.HIS1, his1Δ/his1Δ, arg4Δ/arg4Δ, leu2Δ/leu2Δ, ura3Δ/URA3, iro1Δ/IRO1* | Noble et al., 2010 |
| SN982 | *ssn3****∆∆*** (prototroph) | *ssn3Δ::C.d.HIS1/ssn3Δ::C.m.LEU2, arg4Δ/arg4Δ, leu2Δ/leu2Δ::C.d.ARG4, his1Δ/his1Δ, ura3Δ/URA3, iro1Δ/IRO1* | this study |
| SN978 | *SSN3-*complemented strain | *arg4Δ/arg4Δ, leu2Δ/leu2Δ::SSN3::C.d.ARG4, his1Δ/his1Δ, ssn3Δ::C.d.HIS1/ssn3Δ::C.m.LEU2, ura3Δ/URA3, iro1Δ/IRO1* | this study |
| SN742 | *SFU1*^OE^ | *arg4Δ/arg4Δ, leu2Δ/leu2Δ::C.d.ARG4-TDH3p-SFU1, his1Δ/his1Δ, ura3Δ/URA3, iro1Δ/IRO1, sfu1Δ::C.d.HIS1/sfu1Δ::C.m.LEU2* | this study |
| SN423 | *SEF1-Myc/ SEF1* | *SEF1-13xMyc/SEF1, his1Δ/his1Δ, arg4Δ/arg4Δ, leu2Δ::C.d.LEU2/leu2Δ::C.m.HIS1, ura3Δ/URA3, iro1Δ/IRO1* | Chen et al., 2011 |
| SN646 | *SFU1-Myc/ SFU1* | *SFU1-13xMyc/SFU1, his1Δ/his1Δ, arg4Δ/arg4Δ, leu2Δ::C.d.LEU2/leu2Δ::C.m.HIS1, ura3Δ/URA3, iro1Δ/IRO1* | Chen et al., 2011 |
| SN920 | *SSN3-Myc/ SFU1* | *SSN3-13xMyc-FLP-SAT1/SSN3, his1Δ/his1Δ, leu2Δ::C.d.LEU2/leu2Δ::C.m.HIS1, arg4Δ/arg4Δ, ura3Δ/URA3, iro1Δ/IRO1* | this study |
| SN804 | *SEF1-TAP/ SEF1* | *SEF1-TAP-FLP-SAT1/SEF1, his1Δ/his1Δ, leu2Δ::C.d.LEU2/leu2Δ::C.m.HIS1, arg4Δ/arg4Δ, ura3Δ/URA3, iro1Δ/IRO1* | this study |
| SN893 | *SFU1-TAP/ SFU1* | *SFU1-TAP-FLP-SAT1/SFU1, his1Δ/his1Δ, leu2Δ::C.d.LEU2/leu2Δ::C.m.HIS1, arg4Δ/arg4Δ, ura3Δ/URA3, iro1Δ/IRO1* | this study |
| SN917 | *SSN3-TAP/SSN3* | *SSN3-TAP-FLP-SAT1-FRT/SSN3, his1Δ/his1Δ, leu2Δ::C.d.LEU2/leu2Δ::C.m.HIS1, arg4Δ/arg4Δ, ura3Δ/URA3, iro1Δ/IRO1* | this study |
| SN702 | *SEF1-Myc/SEF1, sfu1****∆∆*** | *SEF1-13xMyc-FLP-SAT1/SEF1, his1Δ/his1Δ, sfu1Δ::C.m.LEU2/sfu1Δ::C.d.HIS1, arg4Δ/arg4Δ, leu2Δ/leu2Δ, ura3Δ/URA3, iro1Δ/IRO1* | this study |
| SN908 | *SEF1-Myc/SEF1, ssn3****∆∆*** | *SEF1-13xMyc-FLP-SAT1/SEF1, his1Δ/his1Δ, ssn3Δ::C.m.LEU2/ssn3Δ::C.d.HIS1, arg4Δ/arg4Δ, leu2Δ/leu2Δ, ura3Δ/URA3, iro1Δ/IRO1* | this study |
| SN778 | *SEF1-Myc/SEF1, SFU1*^OE^ | *SEF1-13xMyc-FLP-SAT1/SEF1, arg4Δ/arg4Δ, leu2Δ/leu2Δ::C.d.ARG4-TDH3p-SFU1, his1Δ/his1Δ, sfu1Δ::C.d.HIS1/sfu1Δ::C.m.LEU2, ura3Δ/URA3, iro1Δ/IRO1* | this study |
| SN953 | *SEF1-Myc/SEF1, SSN3*^OE^ | *SEF1-13xMyc/SEF1, his1Δ/his1Δ, arg4Δ/arg4Δ, SSN3::NAT1-TDH3p-SSN3/SSN3, ura3Δ/URA3, leu2Δ::C.d.LEU2/leu2Δ::C.m.HIS1, iro1Δ/IRO1* | this study |
| SN952 | *SEF1-Myc*^OE^*/****∆*** | *SEF1::NAT1-TDH3p-SEF1-13xMyc/sef1*Δ*, leu2Δ::C.d.LEU2/leu2Δ::C.m.HIS1, his1Δ/his1Δ, arg4Δ/arg4Δ, ura3Δ/URA3, iro1Δ/IRO1* | this study |
| SN960 | *SEF1-Myc*^OE^*/****∆***, *sfu1****∆∆*** | *SEF1::NAT1-TDH3p-SEF1-13xMyc/sef1*Δ*, sfu1*Δ*::C.m.LEU2/sfu1*Δ*::C.d.HIS1, his1Δ/his1Δ, arg4Δ/arg4Δ, ura3Δ/URA3, iro1Δ/IRO1* | this study |
| SN957 | *SEF1-Myc/SEF1, SFU1*^OE^*, SSN3*^OE^ | *SEF1-13xMyc/SEF1, arg4Δ/arg4Δ, his1Δ/his1Δ, ura3Δ/URA3, leu2Δ/leu2Δ::C.d.ARG4-TDH3p-SFU1, SSN3::NAT1-TDH3p-SSN3/SSN3, iro1Δ/IRO1, sfu1Δ::C.d.HIS1/sfu1Δ::C.m.LEU2* | this study |
| SN977 | *SEF1-Myc/SEF1, SSN3(D325A)*^OE^ | *SEF1-13xMyc/SEF1, arg4Δ/arg4Δ, leu2Δ/leu2Δ::C.d.ARG4-TDH3p-SSN3D325A, his1Δ/his1Δ, ura3Δ/URA3, iro1Δ/IRO1, ssn3Δ::C.d.HIS1/ssn3Δ::C.m.LEU2* | this study |
| SN987 | *SSN3(D325A)-MYC*^OE^ | *arg4Δ/arg4Δ, leu2Δ/leu2Δ::C.d.ARG4-TDH3p-SSN3(D325A)-13xMyc-FLP-SAT1, his1Δ/his1Δ, ssn3Δ::C.d.HIS1/ssn3Δ::C.m.LEU2, ura3Δ/URA3, iro1Δ/IRO1* | this study |
| SN902 | *SEF1-TAP/SEF1, SFU1-Myc/SFU1* | *SFU1-13xMyc/SFU1, SEF1-TAP-FLP-SAT1/SEF1, leu2Δ::C.d.LEU2/leu2Δ::C.m.HIS1, his1Δ/his1Δ, arg4Δ/arg4Δ, ura3Δ/URA3, iro1Δ/IRO1* | this study |
| SN950 | *SFU1-TAP /SFU1, SEF1-Myc/SEF1* | *SEF1-13xMyc/SEF1, SFU1-TAP-FLP-SAT1/SFU1, leu2Δ::C.d.LEU2/leu2Δ::C.m.HIS1, his1Δ/his1Δ, arg4Δ/arg4Δ, ura3Δ/URA3, iro1Δ/IRO1* | this study |
| SN918 | *SSN3-TAP/SSN3, SEF1-Myc/SEF1* | *SEF1-13xMyc/SEF1, SSN3-TAP-FLP-SAT1/SSN3, leu2Δ::C.d.LEU2/leu2Δ::C.m.HIS1, his1Δ/his1Δ, arg4Δ/arg4Δ, ura3Δ/URA3, iro1Δ/IRO1* | this study |
| SN921 | *SEF1-TAP/ SEF1, SSN3-Myc/SSN3* | *SSN3-13xMyc-FLP-SAT1/SSN3, SEF1-TAP/SEF1, leu2Δ::C.d.LEU2/leu2Δ::C.m.HIS1, his1Δ/his1Δ, arg4Δ/arg4Δ, ura3Δ/URA3, iro1Δ/IRO1* | this study |
| SN1028 | *SEF1-Myc/SEF1, TAP^OE^* | *SEF1-13xMyc/SEF1, arg4Δ/arg4Δ, leu2Δ/leu2Δ::NAT1-TDH3p-TAP, his1Δ/his1Δ, ura3Δ/URA3, iro1Δ/IRO1,* | this study |
| SN1029 | *SFU1-Myc/SFU1, TAP^OE^* | *SFU1-13xMyc/SFU1, arg4Δ/arg4Δ, leu2Δ/leu2Δ::NAT1-TDH3p-TAP, his1Δ/his1Δ, ura3Δ/URA3, iro1Δ/IRO1,* | this study |
| SN1030 | *SSN3-Myc/SSN3, TAP^OE^* | *SSN3-13xMyc/SSN3, arg4Δ/arg4Δ, leu2Δ/leu2Δ::NAT1-TDH3p-TAP, his1Δ/his1Δ, ura3Δ/URA3, iro1Δ/IRO1,* | this study |
| SN1031 | *SEF1-Myc/SEF1, ssn3****∆∆****, sfu1****∆****/SFU1* | *SEF1-13xMyc/SEF1, ssn3Δ::C.m.LEU2/ssn3Δ::C.d.HIS1,*  *sfu1Δ::C.d. ARG4/SFU1, his1Δ/his1Δ, arg4Δ/arg4Δ, leu2Δ/leu2Δ, ura3Δ/URA3, iro1Δ/IRO1* | this study |
| SN1032 | *SEF1-Myc/SEF1, ssn3****∆∆****, sfu1****∆∆*** | *SEF1-13xMyc/SEF1, ssn3Δ::C.m.LEU2/ssn3Δ::C.d.HIS1,*  *sfu1Δ::C.d. ARG4/sfu1Δ::FLP-SAT1, his1Δ/his1Δ, arg4Δ/arg4Δ, leu2Δ/leu2Δ, ura3Δ/URA3, iro1Δ/IRO1* | this study |

*C.d. ARG4* denotes *Candida dubliniensis ARG4, C.d. HIS1* denotes *Candida dubliniensis HIS1,* and *C.m. LEU2* denotes *Candida maltosa* *LEU2.*
