# Supplementary material for: Post-Transcriptional Regulation of the Sef1 Transcription Factor Controls the Virulence of Candida albicans in Its Mammalian Host
Source: PLoS Pathog. 2012 Nov 1;8(11):e1002956. doi: 10.1371/journal.ppat.1002956 (PMC3486892; doi:10.1371/journal.ppat.1002956)
Supplement: Table S3 — Primers used in this study. (DOCX) [file ppat.1002956.s011.docx]

**Table S3. Primers used in this Study**

| **Primer Name** | **Purpose** | **Sequence** |
| --- | --- | --- |
| SNO464 | Common primer for *Leu2* 5' flank, forward | GGCGAATTGGAGCTCCACCGCGGTGGCGGCCGCTCTAGAACTAGTGGATCGTTTAAACTTGGTAGATTTACAACTGAAGCCG |
| SNO467 | Common primer for *Leu2* 3' flank, reverse | GTCGACGGTATCGATAAGCTTGATATCGAATTCCTGCAGCCCGGGGGATCGTTTAAACTCGAAAACGATGTTTGCACCACCG |
| SNO300 | Common 5' verification primer for Myc-tagging, reverse | CCGTTAATTAACCCGGGGATC |
| SNO301 | Common 3' verification primer for Myc or TAP-tagging, forward | GGAACTTCAGATCCACTAGTTCTAGAGC |
| SNO1014 | Common 5' verification primer for TAP-tagging, reverse | CTCGCTAGCAGTAGTTGGAATATC |
| SNO983 | Common primer for TAP tag, reverse | CTCGAGGGGGGGCCCGGTACCCAAGCCAGTGTGATGGATATCTGCTCAGGTTGACTTCCCCGCGGAATTCGCGTC |
| SNO897 | Common primer for *FRT-FLP-SAT1-FRT* cassette, forward | GCAGATATCCATCACACTGGCTTGGGTACCGGGCCCCCCCTCGAGGA |
| SNO1188 | Common 5' verification primer for *SEF1*-OE and *SSN3*-OE, reverse | GTATTCTGGGCCTCCATGTC |
| SNO794 | Common 3' verification primer for *SEF1*-OE and *SSN3*-OE, reverse | TGTTTCAACCAACCCACTCAAC |
| SNO824 | Common primer for *TDH3p*, forward | GAGCCCTTTGACTGCTTTCATTACTGTTGCTCCTCGTCGACAACGACTGC |
| SNO825 | Common primer for *CdARG4*, reverse | GCAGTCGTTGTCGACGAGGAGCAACAGTAATGAAAGCAGTCAAAGGGCTC |
| SNO951 | *SEF1*-OE 5' flank, forward | GGCGAATTGGAGCTCCACCGCGGTGGCGGCCGCTCTAGAACTAGTGGATCGTTTAAACGCGTCTTCTACATCTCAGTTTGTC |
| SNO952 | *SEF1*-OE 5' flank, reverse | GGGACGAGGCAAGCTTGATCGTGCAGCTCGACTCAAAATCA |
| SNO953 | *SEF1*-OE *SAT1-TDH3p*, forward | ATTTTGAGTCGAGCTGCACGATCAAGCTTGCCTCGTCCC |
| SNO954 | *SEF1*-OE *SAT1-TDH3p*, reverse | ACCTTTTTCAAACTTCATATTTGAATTCAATTGTGATG |
| SNO955 | *SEF1*-OE 3' flank, forward | CATCACAATTGAATTCAAATATGAAGTTTGAAAAAGGTAA |
| SNO956 | *SEF1*-OE 3' flank, reverse | GTCGACGGTATCGATAAGCTTGATATCGAATTCCTGCAGCCCGGGGGATCGTTTAAACTACATCTTTCACATGGGTTTGG |
| SNO655 | *SEF1*-OE 3' verification, reverse | GTTTAATGGCAAAGTAACGTCACCC |
| SNO473 | *SFU1*-OE *LEU2* 5' flank, reverse | CGATACATTTGCGGTACAGAAATGTGAAAAGGGGAGTATTTCTGGAGTGA |
| SNO474 | *SFU1*-OE *CdARG4*, forward | TCACTCCAGAAATACTCCCCTTTTCACATTTCTGTACCGCAAATGTATCG |
| SNO827 | *SFU1*-OE *TDH3p*, reverse | GGTATTGATTCTGTAGGTGAGGTAGGCATGTTAATTGATTTGAATTCAATTGTGATG |
| SNO826 | *SFU1*-OE *SFU1* ORF, forward | CATCACAATTGAATTCAAATCAATTAACATGCCTACCTCACCTACAGAATCAATACC |
| SNO833 | *SFU1*-OE *SFU1* ORF, reverse | TAAATGCTAACTACTGTATATACTGGATTGTGTATAGTTGTAGAACTTG |
| SNO832 | *SFU1*-OE *Leu2*_3' flank, forward | CAAGTTCTACCAACTATACACAATCCAGTATATACAGTGTTAGCATTTA |
| SNO1371 | *SSN3-*OE 5' flank, forward | GGCGAATTGGAGCTCCACCGCGGTGGCGGCCGCTCTAACTAGTGGATCGTTTAAACAGGAGGATGATTTGGAAAGTACAG |
| SNO1372 | *SSN3-*OE 5' flank, reverse | GGGACGAGGCAAGCTTGATTGAGAGACAAAGAGAGACAAAGAGAG |
| SNO1373 | *SSN3-*OE SAT1-TDH3p, forward | GGGACGAGGCAAGCTTGATTGAGAGACAAAGAGAGACAAAGAGAG |
| SNO1374 | *SSN3-*OE SAT1-TDH3p, reverse | CTAAATGAAGCTGAACTATAACTCATATTTGAATTCAATTGTGATG |
| SNO1375 | *SSN3-*OE 3' flank, forward | CATCACAATTGAATTCAAATATGAGTTATAGTTCAGCTTATTTAG |
| SNO1376 | *SSN3-*OE 3' flank, reverse | GTCGACGGTATCGATAAGCTTGATATCGAATTCCTGCAGCCCGGGGGATCGTTTAAACAGACTTTCCCATAAGTACCAGCAG |
| SNO1377 | *SSN3-*OE 5' verification, forward | CGGAGACTGAATGGGATGATAC |
| SNO1378 | *SSN3-*OE 3' verification, reverse | GCCATCAATACCACTATCATCGTC |
| SNO460 | Sef1-Myc, forward | TTAATAATGATAACCAAGATGACGACTTTTTGGGTTGGTTTGATGTTAATATGATGCAAGAGAAACGGATCCCCGGGTTAATTAACGG |
| SNO461 | Sef1-Myc, reverse | ACTTATTCATTACAAAATCATATTAACATAATTACTAACTATTTACATTCTAATGAGGTAGAATCGGCGGCCGCTCTAGAACTAGTGGAT |
| SNO503 | Sef1-Myc 5' verification | TGAAATCTTTTGATTCCAGCAAACC |
| SNO504 | Sef1-Myc 3' verification | CTTCTATTGTTCCACAAGGTGCCAG |
| SNO1322 | Ssn3-13xMyc or Ssn3-TAP 5' flank, forward | GGCGAATTGGAGCTCCACCGCGGTGGCGGCCGCTCTAACTAGTGGATCGTTTAAACGCAGATCAAGCGTTATTACATCC |
| SNO1323 | Ssn3-13xMyc 5' flank, reverse | GTTCACCGTTAATTAACCCGGGGATCCGCCCACGTTTCTTTCTAATTCCACCAGGT |
| SNO1324 | Ssn3-13xMyc 13xMyc-FLP-SAT1, forward | ACCTGGTGGAATTAGAAAGAAACGTGGGCGGATCCCCGGGTTAATTAACGGTGAAC |
| SNO1325 | Ssn3-13xMyc-FLP-SAT1 or Ssn3-TAP-FLP-SAT1, reverse | GTTGTCTGTGAGGATCATGCAGTACCGCGGCCGCTCTAGAACTAGTGGATC |
| SNO1326 | Ssn3-13xMyc or Ssn3-TAP 3' flank, forward | GATCCACTAGTTCTAGAGCGGCCGCGGTACTGCATGACCTCACAGACAAC |
| SNO1327 | Ssn3-13xMyc or Ssn3-TAP 3' flank, reverse | GTCGACGGTATCGATAAGCTTGATATCGAATTCCTGCAGCCCGGGGGATCGTTTAAACCCATAAATAAATCGTGTTGTTCGTTTAC |
| SNO1328 | Ssn3-13xMyc or Ssn3-TAP 5' verification, forward | GGACACCAACAACTGATATTTGG |
| SNO1329 | Ssn3-13xMyc or Ssn3-TAP 3' verification, reverse | GGTTCATCTTTAGGTGGCAGTTT |
| SNO1330 | Ssn3-TAP 5' flank, reverse | GAAATTCTTTTTCCATCTTCTCTTTGACCCTGACCCTGACCCCCCACGTTTCTTTCTAATTCCACCAGGT |
| SNO1331 | Ssn3-TAP TAP tag, forward | ACCTGGTGGAATTAGAAAGAAACGTGGGGGGTCAGGGTCAGGGTCAAAGAGAAGATGGAAAAAGAATTTC |
| SNO1393 | Ssn3D325A *TDH3p*, reverse | GTTTTCTAAATGAAGCTGAACTATAACTCATGTTAATTGATTTGAATTCAATTGTGATG |
| SNO1394 | Ssn3D325A *SSN3* ORF 5', forward | CATCACAATTGAATTCAAATCAATTAACATGAGTTATAGTTCAGCTTCATTTAGAAAAC |
| SNO1395 | Ssn3D325A *SSN3* ORF (D->A point mutation) 5', reverse | GGACTTTTGAATTTTCTTGCTAATCCCAAGGCTCCAATTTTAACAACTCCTTGTGATG |
| SNO1396 | Ssn3D325A *SSN3* ORF (D->A point mutation) 3', forward | CATCACAAGGAGTTGTTAAAATTGGAGCCTTGGGATTAGCAAGAAAATTCAAAAGTCC |
| SNO1397 | Ssn3D325A *SSN3* ORF 3', reverse | TAAATGCTAACTACTGTATATACTGGGATTGAATGTGGTGGAAGACTGA |
| SNO1398 | Ssn3D325A *Leu2* 3', forward | TCAGTCTTCCACCACATTCAATCCCAGTATATACAGTAGTTAGCATTTA |
| SNO889 | Sef1-TAP 5' flank, forward | GGCGAATTGGAGCTCCACCGCGGTGGCGGCCGCTCTGAACTAGTGGATCGTTTAAACAAGCACCAACTCTTCAGCAGTATCC |
| SNO894 | Sef1-TAP 5' flank, reverse | TTCCATCTTCTCTTTGACCCTGACCCTGACCCTTTCTCTTGCATCATATTAACATC |
| SNO895 | Sef1-TAP TAP tag, forward | TGATGCAAGAGAAAGGGTCAGGGTCAGGGTCAAAGAGAAGATGGAAAAAGAATTTC |
| SNO983 | Sef1-TAP TAP tag, reverse | CTCGAGGGGGGGCCCGGTACCCAAGCCAGTGTGATGGATATCTGCTCAGGTTGACTTCCCCGCGGAATTCGCGTC |
| SNO886 | Sef1-TAP-FLP-SAT1, reverse | TTTACATTCTAATGAGGTAGAATCGGCGGCCGCTCTAGAACTAGTGGATC |
| SNO887 | Sef1-TAP 3' flank, forward | TCCACTAGTTCTAGAGCGGCCGCCGATTCTACCTCATTAGAATGTAAATAG |
| SNO888 | Sef1-TAP 3' flank, reverse | GTCGACGGTATCGATAAGCTTGATATCGAATTCCTGCAGCCCGGGGGATCGTTTAAACGTCCAGGGTTCTCTTGTAAACTTG |
| SNO462 | Sef1-TAP 5' verification, forward | CTTACCTATGCTTGAAGCTACTGG |
| SNO898 | Sef1-TAP 3' verification, reverse | CACAAGGTGCCAGAATATACACAG |
| SNO1086 | Sfu1-TAP_5' flank, forward | GGCGAATTGGAGCTCCACCGCGGTGGCGGCCGCTCTAGAACTAGTGGATCGTTTAAACTCACAACTTCTCCTACTTCACAGTC |
| SNO1379 | Sfu1-TAP 5' flank, reverse | GAAATTCTTTTTCCATCTTCTCTTTGACCCTGACCCTGACCCTCCATTTAACAACTTCCCAATAGAAAG |
| SNO1380 | Sfu1-TAP TAP tag, forward | CTTTCTATTGGGAAGTTGTTAAATGGAGGGTCAGGGTCAGGGTCAAAGAGAAGATGGAAAAAGAATTTC |
| SNO1073 | Sfu1-TAP -FLP-SAT1, reverse | AAGGGGATTGTTTTGCATACTCGGCGGCCGCTCTAGAACTAGTGGATC |
| SNO1074 | Sfu1-TAP 3' flank, forward | TCCACTAGTTCTAGAGCGGCCGCCGAGTATGCAAAACATCCCCTTTC |
| SNO1075 | Sfu1-TAP 3' flank, reverse | GTCGACGGTATCGATAAGCTTGATATCGAATTCCTGCAGCCCGGGGGATCGTTTAAACCAGACGATAACTTGGAAGATTGTG |
| SNO1089 | Sfu1-TAP 5' verification, forward | CGCCATATAGTGGATCTGGAAG |
| SNO1085 | Sfu1-TAP 3' verification, reverse | AGGACGTAATGATGATGATGAAGG |
| SNO1416 | *SSN3* complement-*LEU2* 5' flank, reverse | CATTCACAAGGGATAATTCATCGTGGCAGCGAACTTACACCACTCGAAAAGGGGAGTATTTCTGGAGTGAA |
| SNO1417 | *SSN3* complement-*SSN3* genomic locus, forward | TCACTCCAGAAATACTCCCCTTTTCGAGTGGTGTAAGTTCGCTGCCACGATGAATTATCCCTTGTGAATG |
| SNO1418 | *SSN3* complement-*SSN3* genomic locus, reverse | CGATACATTTGCGGTACAGAAATGTGCATTGGAGAGACTTGGTGAGTTAC |
| SNO1419 | *SSN3* complement-*CdARG4*, forward | GTAACTCACCAAGTCTCTCCAATGCACATTTCTGTACCGCAAATGTATCG |
| SNO469 | *SSN3* complement-*CdARG4*, reverse | TAAATGCTAACTACTGTATATACTGAGTAATGAAAGCAGTCAAAGGGCTC |
| SNO466 | SSN3 complement-*LEU2* 3' flank, forward | GAGCCCTTTGACTGCTTTCATTACTCAGTATATACAGTAGTTAGCATTTA |
| SNO819 | qPCR for *ACT1* ORF, forward | GTG GTA CTA CCA TGT TCC CAG G |
| SNO820 | qPCR for *ACT1* ORF, reverse | GAT AGA ACC ACC AAT CCA GAC AGA G |
| SNO654 | qPCR for *SEF1* ORF, forward | TCCACAACGTGCATTGTCATATACG |
| SNO655 | qPCR for *SEF1* ORF, reverse | GTTTAATGGCAAAGTAACGTCACCC |
| SNO656 | qPCR for *SFU1* ORF, forward | GATGTAATGGTACTGGAGGATCGGC |
| SNO657 | qPCR for *SFU1* ORF, reverse | AGAGCTTTCATCTTCAGTAGTAGCG |
| SNO1311 | qPCR for *SSN3* ORF, forward | ACTAGAGCTTCGATTCCTCAACC |
| SNO1312 | qPCR for *SSN3* ORF, reverse | CCCATAAGTACCAGCAGCAATA |
| SNO1550 | *TAP-*OE 5’ flank, reverse | GGGGACGAGGCAAGCTTGATGAAAAGGGGAGTATTTCTGGAGTGAA |
| SNO1551 | *TAP-*OE *SAT1-TDH3p* cassette, forward | TTCACTCCAGAAATACTCCCCTTTTCATCAAGCTTGCCTCGTCCCC |
| SNO1552 | *TAP-*OE *SAT1-TDH3p* cassette, reverse | GAAATTCTTTTTCCATCTTCTCTTTTCCATATTTGAATTCAATTGTGATG |
| SNO1553 | *TAP-*OE, forward | CATCACAATTGAATTCAAATATGGAAAAGAGAAGATGGAAAAAGAATTTC |
| SNO1554 | *TAP-*OE, reverse | TAAATGCTAACTACTGTATATACTGTCAGGTTGACTTCCCCGCGGAATTCGCGTC |
| SNO1555 | *TAP-*OE 3’ flank, forward | GACGCGAATTCCGCGGGGAAGTCAACCTGACAGTATATACAGTAGTTAGCATTTA |
