# Supplementary material for: Post-Transcriptional Regulation of the Sef1 Transcription Factor Controls the Virulence of Candida albicans in Its Mammalian Host
Source: PLoS Pathog. 2012 Nov 1;8(11):e1002956. doi: 10.1371/journal.ppat.1002956 (PMC3486892; doi:10.1371/journal.ppat.1002956)
Supplement: Table S4 — Plasmids used in this study. (DOCX) [file ppat.1002956.s012.docx]

**Table S4. Plasmids Used in this Study**

| **Plasmid** | **Insert** | **Use** | **Selectable Markers** | **Vector** |
| --- | --- | --- | --- | --- |
| pSN147 | *PmeI-SEF1* promoter fragment*-SAT1-TDH3* promoter*-SEF1* ORF fragment*-PmeI* | *SEF1* overexpression | AmpR,  *ScURA3*,  NatR | pRS316 |
| pSN141 | *PmeI-SFU1* promoter fragment*-CdARG4-TDH3* promoter*-SFU1* ORF fragment-*PmeI* | *SFU1* overexpression | AmpR, *ScURA3*,  *CdARG4* | pRS316 |
| pSN229 | *PmeI-SSN3* promoter fragment *SAT1*- *TDH3* promoter*-SSN3* ORF fragment*-PmeI* | *SSN3* overexpression | AmpR, *ScURA3*,  NatR | pRS316 |
| pSN239 | *PmeI-LEU2* upstream sequence-*CdARG4-TDH3p-SSN3D325A-LEU2* downstream sequence-*PmeI* | *SSN3* kinase dead | AmpR, *ScURA3*,  *CdARG4* | pRS316 |
| pSN218 | *PmeI-SSN3* C-terminal ORF sequence*-13xMyc-FRT-FLP-SAT1-FRT-SSN3* downstream sequence*-PmeI* | Myc-tagged Ssn3 | AmpR, *ScURA3*,  NatR | pRS316 |
| pSN150 | *PmeI-SEF1* C-terminal ORF sequence*-TAP-FRT-FLP-SAT1-FRT- SEF1* downstream sequence*-PmeI* | TAP-tagged Sef1 | AmpR, *ScURA3*,  NatR | pRS316 |
| pSN228 | *PmeI-SFU1* C-terminal ORF sequence*-TAP-FRT-FLP-SAT1-FRT-SFU1* downstream sequence*-PmeI* | TAP-tagged Sfu1 | AmpR, *ScURA3*,  NatR | pRS316 |
| pSN219 | *PmeI-SSN3* C-terminal ORF sequence*-TAP-FRT-FLP-SAT1-FRT-SSN3* downstream sequence*-PmeI* | TAP-tagged Ssn3 | AmpR, *ScURA3*,  NatR | pRS316 |
| pSN273 | *Pme1-SSN3* C-terminal ORF sequence*-13xMyc-FRT-FLP-SAT1-FRT-SSN3* downstream sequence*-PmeI* | Myc-tagged SSN3 kinase dead | AmpR, *ScURA3*,  NatR | pRS316 |
| pSN292 | *PmeI-LEU3* upstream sequence-*FRT-FLP-SAT1-FRT-TDH3 promoter-TAP-LEU2* downstream sequence-*PmeI* | TAP tag overexpression | AmpR,  *ScURA3*,  NatR | pRS316 |
| pSN184 | *PmeI-SFU1 upstream flank-FRT-FLP-SAT1-FRT-SFU1 downstream flank-PmeI* | *SFU1* Knockout | AmpR,  *ScURA3*,  NatR | pRS316 |

*PmeI* denotes the recognition sequence for this restriction enzyme; *CdARG4* denotes the *ARG4* gene from *Candida dubliniensis*; *ScURA3* denotes the *URA3* gene from *Saccharomyces cerevisiae,* AmpR denotes resistance to ampicillin, NatR denotes resistance to nourseothricin.
